# Energy and nutrient production in Ethiopia, 2011-2015: Implications to supporting healthy diets and food systems

**DOI:** 10.1371/journal.pone.0213182

**Published:** 2019-03-12

**Authors:** Kaleab Baye, Kalle Hirvonen, Mekdim Dereje, Roseline Remans

**Affiliations:** 1 Center for Food Science and Nutrition, College of Natural Sciences, Addis Ababa University, Addis Ababa, Ethiopia; 2 Bioversity International, Addis Ababa, Ethiopia; 3 Development Strategy and Governance Division, International Food Policy Research Institute, Addis Ababa, Ethiopia; 4 Center for Development Research, University of Bonn, Bonn, Germany; 5 Bioversity International, Heverlee, Belgium; 6 Department of Agrotechnology and Food Sciences, Wageningen University & Research, Wageningen, the Netherlands; Kansas State University, UNITED STATES

## Abstract

Agricultural sector plays a key role towards achieving healthier diets that are deemed critical for improving health and nutritional outcomes. To what extent the current food supply systems support healthy diets remains unknown. Using annual and nationally representative data on crop and livestock production in Ethiopia, we assess the national agricultural sector from a nutrition lens and its role in supporting healthy diets in the country. We do so by converting the agricultural production into energy and nutrients for the period of 2011–2015. These data show that the national food production has increased dramatically over the 5-year period to supply more than 3,000 calories per capita in 2015. Moreover, nutrient production gaps have substantially decreased (2011–15), but deficits in energy (5%), vitamin C (16%), and calcium (9%) production remained in 2015. However, this production growth–coming primarily from the cereal sector and at the expense of other food groups–led to a decrease in production diversity as reflected by a drop in the Shannon index between 2011 and 2015. Together these findings imply that the production increases in Ethiopia would need to be sustained to feed the rapidly growing population but more emphasis should be given to diversification to support healthy and nutritionally diversified diets.

## Introduction

One in three people worldwide is affected by one or more forms of malnutrition. Nearly 800 million are undernourished, 1.9 billion adults are overweight or obese [1]. About two billion people worldwide are anemic and suffer from micronutrient deficiencies [2]. Globally, three billion people have poor quality diets. Poor diets are now considered as the leading causes of morbidity and mortality putting people at a greater risk than unsafe sex, tobacco, drug and alcohol use combined [3]. Although what constitutes a healthy diet is context-dependent, there is a strong evidence that diverse diets which include nutrient-dense foods such as whole grains, fruits, vegetables, legumes and nuts along with moderate consumption of animal source foods are associated with healthier outcomes, ranging from decreased risk of heart disease, cancer and obesity [3–5].

What people choose to eat is determined by various factors including consumer preference, the food environment which includes affordability and accessibility, and the food supply system that consists of production, storage and transportation, processing, and marketing; altogether referred as the *food system* [6]. Therefore, even when the most effective nutrition-specific interventions that target the immediate causes of malnutrition are applied, only 26 percent of the malnutrition cases can be averted [7]. Thus, complementary nutrition-sensitive interventions that target the underlying causes of malnutrition are key [8]. More importantly, there is a need to move towards a food system that not only feeds, but also nourishes its people [9].

In many low- and middle-income countries, domestic agricultural production dictates the kind of food that is available and accessible for consumption [10]. This is particularly the case for sub-Saharan Africa where there is still relatively limited international trade in agricultural products [11]. Agricultural policies in Africa, much like in other continents, have focused on productivity, measured as grain yield of major cereal crops like maize or in kilocalories available for the population [12, 13]. It is only recently that nutrition–in its broadest sense (i.e. diet quality)–has emerged in agricultural policy discourses. Many African countries are in the process of drafting nutrition-sensitive agriculture strategies that aim to increase the availability of nutrient-dense foods [14, 15]. However, little information exists on how the agricultural sector has been performing from the perspective of supporting healthier diets through the production of nutrient-dense foods. As outlined in a recent commentary published in *Nature* [9], such evidence is urgently needed to inform nutrition-sensitive agriculture strategies on how to support healthy diets.

In this paper, using nationally representative data for Ethiopia, we take a first step towards evaluating the role of agricultural production in supporting healthy diets and improved nutrition. Ethiopia provides an excellent location for this case study. First, Ethiopian children consume one of the least diversified diets in sub-Saharan Africa [16]. In 2016, only 14 percent of Ethiopian children age 6–23 months met the WHO criteria for a minimum dietary diversity; at least four food groups out of seven [17]. Second, with little international trade in food products, the domestic agricultural sector is responsible for feeding the 2^nd^ largest population in Africa, nearly 90 million people. Third, the Ethiopian agricultural sector has performed remarkably well in increasing the cereal production and productivity in the country [18]. However, there is no evidence on the performance of the agricultural sector in supplying nutrient-dense foods to close nutrient and energy gaps. Fourth, the country has recently drafted its first nutrition-sensitive agriculture strategy [19], which provides a unique opportunity to shape the agricultural sector to support healthy diets.

Therefore, the present study evaluates the performance of the domestic agricultural sector towards supporting healthier diets at the national and regional level in Ethiopia. To this end, we evaluate the diversity of the agricultural food supply as well as its energy and nutrient supply from 2011–2015.

## Methods

### Data on crop and livestock production

The data for this study come from the Central Statistical Agency (CSA) of Ethiopia. We used the CSA's Agricultural Sample Survey reports to estimate annual agricultural production in the country. More specifically, we relied on CSA's three annual flagship reports. The *Report on Area and Production of Major Crops*, *private peasant holdings* provides the total annual crop output in 10 out of the 11 administrative regions of the country. Only the production in the capital, Addis Ababa, is not reported. The crop output for *meher* (long rainy season and the main cropping season) and *belg* (short rainy season) are reported separately. The *Report on Livestock and Livestock Characteristics*, *private peasant holdings* provides the annual estimates of the livestock population and livestock production. The sample sizes are large–typically containing more than 40,000 rural households. These reports are based on nationally representative data collected by the CSA each year, and are also used by the Food and Agricultural Organization (FAO) to generate the widely used Food Balance Sheets (FBS) for Ethiopia [20]. The advantage of using the original sources, rather than the FBS, is that it permits to study food production at sub-national level and to convert production into a number of micronutrients that are not reported in FBS.

We digitalized these reports. For crop output, we aggregated the total annual production for each crop in each region during *meher* and *belg* seasons as well as the total crop output produced by the commercial sub-sector. For livestock products, we took the total annual milk (cow and camel), eggs and honey produced. The CSA reports give the number of different livestock types slaughtered. These numbers were converted to kilograms of beef, sheep, goat, camel and poultry meat using conversion factors to account for edible portions only [21].

### Production diversity

Although various dietary diversity measurement indicators exist, the WHO recommended seven-food group dietary diversity score was used for this study [22]. This dietary diversity score was consistently found to be a good predictor of energy and nutrient adequacy of children’s diets and stunting [23–25]. Accordingly, foods were categorized into seven groups: 1) Grains, roots and tubers; 2) Legumes and nuts; 3) Dairy products; 4) Poultry, fish, meat; 5) Eggs; 6) Vitamin A-rich fruits and vegetables; and 7) Other fruits and vegetables. To capture the remaining foods like fenugreek, red peppers, garlic, coffee, sugarcane and honey, we have added an additional food group and called it ‘other foods’.

We measured the overall diversity of the food production using the Shannon index. The Shannon diversity index (H) is typically used in Ecology to characterize species diversity in a given area. The Shannon's index considers both richness (= number of species) and abundance (= how much of each species is present as proportion of the whole population) of the species and was calculated as follows:

$$H=-\sum_{i=1}^{7}p_{i}ln(p_{i}),$$

where, *p_i_* is the proportion of the total production coming from food group *i*. We only considered the 7-food groups and ignored the ‘Other foods’ category that was not nutritionally relevant when computing the Shannon Index. Larger Shannon index values reflect higher diversity, whereas values close to zero indicate very low diversity.

### Energy and nutrient contribution of the food supplied by agricultural production

The annual crop and livestock production was then converted into energy and 9 different micro and macro nutrients using the Ethiopian food composition tables [26]. The choice of these nutrients was based on their biological importance (e.g. physical and cognitive development of children) as well as the availability of reliable composition data. We used FAO reject estimates which corresponds to weight estimations of edible portions of foods produced to calculate energy and nutrients supplied. The total energy and nutrients were divided by the total population for that specific year reported in Table A in S1 File. The energy and nutrients produced were expressed on a per day basis by dividing by 365 to enable comparison with daily requirements. It is, however, worth noting that agricultural production in Ethiopia is largely rain-fed [27], and therefore highly seasonal.

### Energy and nutrient requirements

To estimate dietary requirements, a national Estimated Average Requirement (EAR) was calculated for each nutrient using data from WHO [28] and the Institute of Medicine [29, 30]. The EARs were derived from the reference nutrient intake (RNI), which is the intake level sufficient for approximately 97 percent of a specific sex and life-stage group. RNIs provided by WHO and IOM [28–30] were converted to EARs using standard conversion factors [31].

We calculated the sex and life-stage specific EARs, using national population estimates disaggregated by sex and age [32]. Crude pregnancy rate was calculated for each age group as crude birth rate (CBR) x 280/365, assuming pregnancy lasts for 280 days. The proportion of lactating women was calculated by assuming that breastfeeding was continued until two years (CBR x 2), as previously described in Joy et al., [33].

The EAR for calcium was set by assuming low-animal protein intake relative to Western intake levels [21]. The EARs for iron (adult female EAR = 13.4 mg day^−1^) and Zn (adult female EAR = 8.2 mg day^−1^) assumed low bioavailability (10% for iron and 15% for zinc) [28]. Because of hemostatic response during pregnancy, an increase of 50% in iron bioavailability was assumed (from 10 to 15%). Pregnant women were therefore assumed to require additional 20 mg iron/day. A total net Fe requirement of 840 mg was thus assumed for pregnant women [28]. The Zn EAR during pregnancy was estimated to be 12.5 mg/day. For lactating women, the EAR for Zn was the weighted average of 0–3, 3–6 and 6+ months, assuming two years of lactation (i.e. 12.8 mg day^−1^). For pregnant and lactating adolescents (aged 15–19 years), the Zn EARs during both the third trimester of pregnancy and 0–3 months of lactation periods were assumed to be double to those of non-pregnant adolescents of the same age group [28].

### Estimating intake distribution and prevalence of nutrient production deficits

We used the EAR cut-off point method to estimate the prevalence of food production deficits [34]. For each nutrient, we estimated a population distribution around the mean estimated intake per capita derived from the food production data. This was done by calculating a coefficient of variation (CV) of intake based on within-subject variation values obtained from published literature [35]. The CVs used were as follows: Energy/protein/zinc/calcium/niacin (CV = 0.25), vitamin A (CV = 0.45), vitamin C/iron (CV = 0.4), thiamin/riboflavin (CV = 0.3). A nutrient intake distribution was assumed to be normally distributed for CV values 0.3 or lower and log-normally if the CV was greater than 0.3. We applied this CV to obtain a distribution of estimated micronutrient intakes across each year. The proportion of the population below the national EAR was considered to estimate the production deficits.

## Results

Table 1 shows how the total food production in Ethiopia increased by 56 percent from 301 million quintals in 2011 to 469 million quintals in 2015. We also see that the three largest regions in terms of population and arable land–Oromia, SNNP and Amhara–together produce more than 90 percent of the annual food production in the country. A closer look at the total food production by food group is provided in Table 2. This reveals that apart from dairy and flesh foods, the production of all plant-based food groups has considerably increased over the five-year period. Staple crop production increased the most (12 percent/year), while other plant-based food groups increased between 3.7 and 7.6 percent per annum. Apart from eggs, the production of animal source foods remained stagnant.

**Table 1:** Total Food production in millions of quintals (and % of total by region), 2011–2015.

	2011	2012	2013	2014	2015
National	300.7	317.7	373.7	447.1	469.3
*As a % of the National*:					
Oromia	45.8	45.8	43.3	42.4	42.6
SNNP	18.6	19.2	24.9	29.2	28.4
Amhara	26.5	26.1	23.8	21.5	21.7
Tigray	5.7	5.4	4.9	4.1	4.4
BG	1.4	1.5	1.3	1.1	1.1
Somali	0.8	0.9	0.8	0.7	0.7
Afar	0.7	0.8	0.7	0.6	0.6
Gambella	0.4	0.2	0.2	0.2	0.2
Dire Dawa	0.1	0.1	0.1	0.1	0.1
Harar	0.1	0.1	0.1	0.1	0.1

Source: authors' calculations from CSA's Agricultural Sample Survey reports.

Note: SNNP = Southern Nations, Nationalities, and Peoples' Region, BG = Benishangul-Gumuz.

**Table 2:** Food production in millions of quintals by food group, 2011–2015.

Food categories	2011	2012	2013	2014	2015	CAGR
Grains, roots and tubers	213.9	225.6	275.5	349.7	368.2	11.6
Legumes and nuts	29.9	33.3	38.9	39.8	38.4	5.8
Dairy products	33.0	29.6	32.5	30.5	32.4	-0.4
Poultry, fish, meat	1.53	1.55	1.36	1.40	1.48	-0.7
Eggs	0.68	0.60	0.68	0.73	0.86	4.9
Vitamin A rich fruits and vegetables	1.21	1.31	1.15	1.11	1.50	4.4
Other fruits and vegetables	14.8	15.4	14.7	14.9	17.1	7.6
Other foods	19.1	18.3	20.3	22.9	23.0	3.7
**Total**	**300.7**	**317.7**	**373.7**	**447.1**	**469.3**	**9.3**

Source: authors' calculations from CSA's Agricultural Sample Survey reports.

Note: CAGR = Compound Annual Growth Rate.

Table 3 shows the trends with respect to production diversity; Shannon Index. Despite increases in total volume, the production diversity decreased by 3.6 percent per annum. The fall is particularly marked in the 2^nd^ most important production region of the country (see Table 1), SNNP where the Shannon index fell from 1.34 to 0.91 between 2011 and 2015 (or by 7.4 percent per annum). In contrast, in Afar, the production diversity increased substantially over the five-year period. However, in 2015, Afar still has the lowest production diversity compared to other regions.

**Table 3:** Shannon diversity index 2011–2015, by region.

	2011	2012	2013	2014	2015	CAGR
National	1.07	1.07	1.00	0.89	0.89	-3.6
Oromia	0.99	1.01	0.95	0.87	0.84	-3.2
SNNP	1.34	1.26	1.06	0.84	0.91	-7.4
Amhara	0.91	0.95	0.94	0.86	0.83	-2.0
Tigray	0.83	0.80	0.81	0.75	0.78	-1.1
BG	0.91	0.97	0.97	0.92	0.89	-0.4
Somali	0.86	0.98	0.79	0.94	0.87	0.2
Afar	0.16	0.71	0.63	0.58	0.63	31.5
Gambella	1.39	1.36	1.40	1.35	1.20	-2.9
Dire Dawa	1.00	1.18	1.12	1.07	1.00	0.0
Harar	0.99	0.84	1.06	1.07	1.55	9.3

Source: authors' calculations from CSA's Agricultural Sample Survey reports.

Note: CAGR = Compound Annual Growth Rate. Note: SNNP = Southern Nations, Nationalities, and Peoples' Region, BG = Benishangul-Gumuz.

In Table 4 we assess the *per capita* production of energy and nutrients. In line with Table 1, we see substantial improvements in energy and nutrient production. Moreover, energy and nutrient production gaps at the national level were widespread in 2011, as shown by extremely high prevalence of estimated gaps. In contrast, only gaps in energy, vitamin C and calcium production remained in 2015. A regional disaggregation provided in Table B in S1 File shows that high prevalence of energy and nutrient production deficits in 2015 were estimated for Somali, Afar, and Gambella region.

**Table 4:** Time trends in energy and nutrient production per capita, 2011–2015.

	2011	2012	2013	2014	2015	EAR
Energy (kcal)	2,686.9 (37.5)	2,766.8 (32.7)	3,011.4 (20.0)	3,455.6 (6.0)	3,503.8 (5.1)	2,488.4
Protein (g)	70.0 (0.7)	72.5 (0.3)	77.3 (0.1)	82.3 (0.0)	84.7 (0.0)	43.2
Vitamin A (RAE)	204.6 (84.9)	165.3(89.6)	289.4(70.5)	379.4 (50.6)	492.0(26.1)	382.1
Ascorbic acid (mg)	29.8 (60.2)	32.5 (52.1)	40.8 (28.4)	42.3 (24.7)	46.2 (16.4)	33.2
Iron (mg)	142.2 (0.0)	152.6(0.0)	166.1(0.0)	192.6(0.0)	190.0(0.0)	10.3
Zinc (mg)	17.1(0.4)	17.6(0.2)	18.7 (0.1)	20.1(0.0)	20.8(0.0)	10.3
Calcium (mg)	731.3 (32.9)	696.5 (39.6)	761.4 (27.5)	909.5 (8.6)	904.3 (9.1)	645.4
Thiamine (mg)	21.9 (0.0)	22.2(0.0)	23.3(0.0)	26.1(0.0)	27.2(0.0)	0.8
Niacin (mg)	20.4(0.0)	21.4(0.0)	22.3(0.0)	23.5(0.0)	25.0(0.0)	10.0
Riboflavin (mg)	1.2 (12.1)	1.2 (9.8)	1.4 (3.5)	1.6 (0.5)	1.6(0.4)	0.9

Source: authors' calculations from CSA's Agricultural Sample Survey reports.

Note: EAR = estimated average requirement; values are production, values in parentheses are percent of produced relative to EAR.

**Fig 1:**
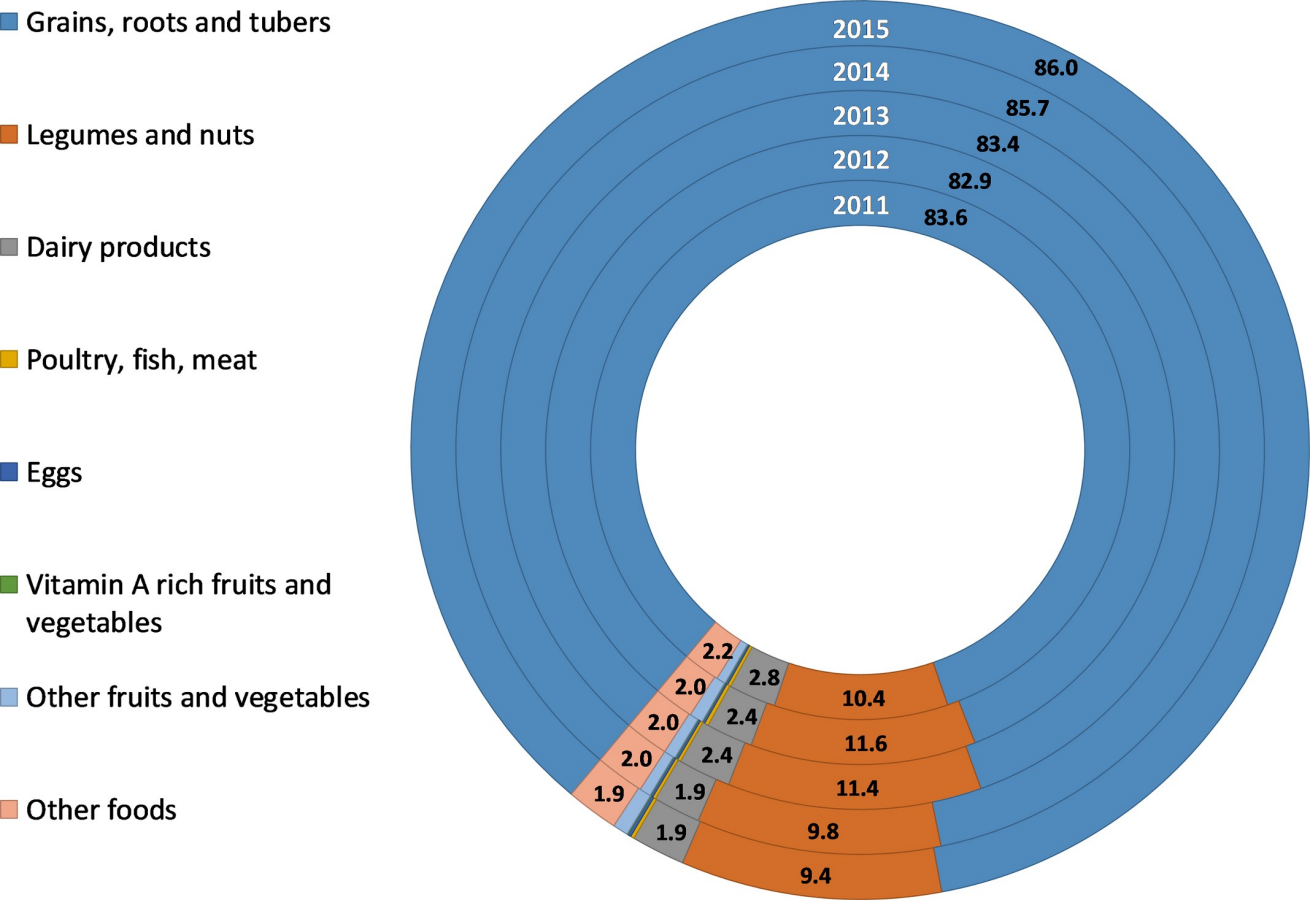
Food groups’ contribution to energy supply from agricultural production. Note: Food groups whose values are not labeled represent <1% of the energy intake.

Finally, Fig 1 disaggregates the energy supply by each food group. As expected, the staple food group (Cereals, starchy roots and tubers) accounted for most of the energy produced (> 80%) in the country. Pulses contributed about 10 percent of the total energy, and showed slight decrease over the 2011–2015 period. Dairy contributed less than 3 percent of the total energy in 2011, and this figure dropped to 1.9 percent in 2015. Eggs, meat, fish and poultry, and fruits and vegetables including vitamin A-rich ones contributed to less than 1 percent of the total energy produced. Meanwhile, there were little to no imports of these food groups (see Figure A in S1 File).

## Discussion

The present study indicates that Ethiopia’s food production sector has grown substantially between 2011 and 2015. Importantly for food security, this increase in production has kept pace with the rapidly growing population over the same period. The estimated energy and nutrient gaps decreased over the period, but mainly because of increases in the production of starchy staples. Consequently, a considerable decrease in the diversity of food production was observed.

In line with Ethiopia’s agricultural transformation plan (2011–2015), the national food production has increased dramatically to supply ≥3,000 kcal/capita in 2015. This figure is not accounting for post-harvest losses due to lack of accurate data; however, the high energy production (~3,500 kcal/capita) suggests that even with substantial post-harvest loss, the energy supplied may still be sufficient to meet per capita energy requirements. This finding is in contrast with studies that argue that current production levels are not sufficient [36–38]. However, food production is unequally distributed across the country and not all regions in Ethiopia are producing enough to feed its people. While some regions like Oromia and Amhara were over-producers, other administrative regions like Afar and Somali heavily rely on the food produced in other regions, partly because of more constrained agro-ecological conditions. This highlights the need of paying attention to marketing, storage, transport, and processing sub-systems of the food supply to ensure well-functioning and well-connected food systems [39].

Agriculture should not only feed, but also nourish its people. The increased production will increase the availability of not only energy but also nutrients. This is reflected by the decline in the number and magnitude of nutrient gaps estimated from the food supply. However, the increases in produced output were highly uneven across different food groups. Cereals and starchy staples dominated the total production, while the production of more nutrient-dense foods like dairy and animal-source foods declined over the study period. This reflects the cereal yield focused agricultural policy that has prevailed in many African countries including Ethiopia [12, 13].

Although the nutrients supplied increased with increased cereal production, this does not necessarily translate into reductions in nutrient deficiencies observed at the population level. Cereals contain nutrients in low densities, and therefore meeting the nutrient requirements would require individuals to consume unrealistic amounts of cereals. For example, the production data reported here suggests that the country produces sufficient amount of zinc while the results from the recent Ethiopian national food consumption survey that used quantitative 24h recall indicate that a large share of the population (50.4%) are zinc deficient [40]. Moving away from nutrients to diets, such a monotonous production will likely contribute to monotonous diets–with limited potential to improve child growth outcomes [41]. Moreover, such monotonous diets can also contribute to the increasing burden of chronic diseases. The recently published Prospective Urban Rural Epidemiology (PURE) study showed increased overall mortality and higher risk of major cardiovascular diseases when energy intake from carbohydrate exceeded 60–70% [42]. According to the Ethiopian food consumption survey, 60–80% of the energy intake of children and adults is from carbohydrates [43] while cardiovascular diseases were among the top causes of premature mortality in Ethiopia in 2015 [43]. Together these findings provide suggestive evidence of the link established in the PURE study.

There has been an active debate of whether market-based solution or diverse production strategies are better for improving diets in sub-Saharan Africa [44–48]. Although these policy options have their own merits, it seems clear that a national food supply that is diverse enough to support dietary diversity at the individual level is critical. As clearly illustrated in this study, for regions with limited agricultural production (e.g. Afar), deepening inter-regional trade would seem a more feasible strategy to improve diets than increasing and diversifying region's own production. However, the overall production in the country is of limited diversity, and thus even if cross-regional trade within the country is strengthened, there is not enough production diversity at the national level to support dietary diversity [47]. Besides, this gap is unlikely to be filled through imports, at least in the short-term, as the overall contribution of imports is currently extremely low, particularly for nutrient rich foods (see Figure A in S1 File).

The declining production diversity witnessed over the 2011–2015 period is a cause for concern. The production of nutrient-dense food groups was not growing at the same pace as cereals. This finding is supported by a recent research from Ethiopia that found that the real prices of nutrient-dense food have been rising considerably faster than general inflation, or the prices of staple foods in the past decade (2007–2016) [49]. The government of Ethiopia (through its health extension program) and various non-governmental organizations are implementing nutrition-specific and sensitive interventions that aim to improve the caregivers' knowledge about the health benefits of nutritious diets [50–53]. However, based on the evidence presented in this study but also previously [54], it seems clear that the success of these interventions will be limited if the inadequate supply of nutrient-dense foods is not addressed simultaneously. Overall, this highlights the need for food systems-wide interventions that not only focus on consumer behavioral change, but also influence the food environment (access and availability) and the food supply towards supporting a healthy diet.

Several limitations need to be considered when interpreting our results. First, we did not consider the role of international trade in agricultural products. However, we expect that this omission has negligible impact on the estimates reported here. Exports form mere 2 percent of the total domestic production (Figure A panel A in S1 File). Moreover, less than 4 percent of the domestic food supply comes from imports, out of which more than 95 percent are cereals (Figure A panel B in S1 File). As a result, our estimates are likely to be slightly underestimated, especially for the energy produced in the country. Second, our estimates consider the agricultural production, which does not necessarily equate to consumption. Factors like post-harvest losses, food waste, seasonality of food production, food prices, food provision, preparation, and intra-household distribution can all affect what is actually consumed from what is produced. Nevertheless, this exercise has the advantage of indicating what could be achieved with better market integration, more efficient post-harvest handling, storage and processing infrastructures along with improved consumer behavior.

Notwithstanding the above limitations, by analyzing a nationally representative data collected annually in over 40,000 households across the country, our study has for the first time showed that Ethiopia’s energy and nutrition production has increased dramatically over the past years’ but that this increase was mainly from cereal production that came at a cost of lower production diversity. The present study highlights that while sustaining the increases in national agricultural production is key to feed its people, this production need to be diversified to support healthy diets.

## Supporting information

S1 File Population by region, 2011–2015 (Table A). Prevalence (%) of energy and nutrient gaps relative to population-adjusted estimated average requirements by region, 2015 (Table B). Food supplied (%) from production, import and export (Panel A) and import and export (x 1000 metric tons) by food groups (Panel B), 2011–2013 (Figure A).
(DOCX)
