# Supplementary material for: Energy and nutrient production in Ethiopia, 2011-2015: Implications to supporting healthy diets and food systems
Source: PLoS One. 2019 Mar 12;14(3):e0213182. doi: 10.1371/journal.pone.0213182 (PMC6413914; doi:10.1371/journal.pone.0213182)
Supplement: S1 File — Population by region, 2011–2015 (Table A). Prevalence (%) of energy and nutrient gaps relative to population-adjusted estimated average requirements by region, 2015 (Table B). Food supplied (%) from production, import and export (Panel A) and import and export (x 1000 metric tons) by food groups (Panel B), 2011–2013 (Figure A). (DOCX) [file pone.0213182.s001.docx]

**Supporting Information**

**Energy and nutrient production in Ethiopia, 2011-2015: implications to supporting healthy diets and food systems**

Kaleab Baye, Kalle Hirvonen, Mekdim Dereje, Roseline Remans

**Table A: Population by region, 2011-2015**

| **Region** | **2011** | **2012** | **2013** | **2014** | **2015** |
| --- | --- | --- | --- | --- | --- |
| Tigray | 4,661,187 | 4,760,947 | 4,860,574 | 4,960,003 | 5,055,999 |
| Afar | 1,534,396 | 1,582,848 | 1,630,767 | 1,678,000 | 1,723,007 |
| Amhara | 18,814,075 | 19,221,033 | 19,622,902 | 20,018,988 | 20,399,004 |
| Oromia | 30,171,257 | 31,042,386 | 31,924,133 | 32,815,995 | 33,691,991 |
| Somali | 4,872,821 | 5,014,830 | 5,159,558 | 5,307,002 | 5,452,994 |
| BG | 894,857 | 920,545 | 947,567 | 975,998 | 1,005,001 |
| SNNP | 16,522,833 | 16,954,155 | 17,392,242 | 17,837,005 | 18,276,012 |
| Gambella | 358,634 | 370,542 | 382,997 | 396,000 | 409,002 |
| Harar | 207,479 | 213,648 | 219,825 | 226,000 | 232,000 |
| Dire Dawa | 387,073 | 400,310 | 413,626 | 427,000 | 440,000 |
| Addis Ababa | 2,967,091 | 3,041,118 | 3,117,070 | 3,194,999 | 3,273,001 |
| **Total** | **81,391,703** | **83,522,362** | **85,671,261** | **87,836,990** | **89,958,011** |

*Source: CSA 2013. Population Projection of Ethiopia for All Regions at Woreda Level from 2014 – 2017. Central Statistical Agency (CSA) of Ethiopia: Addis Ababa*

*Note: SNNP= Southern Nations, Nationalities, and Peoples' Region, BG = Benishangul-Gumuz*

**Table B Prevalence (%) of energy and nutrient gaps relative to population-adjusted estimated average requirements by region, 2015**

|  | **Oromia** | **Amhara** | **SNNP** | **Somali** | **Tigray** | **Afar** | **BG** | **Gambella** |
| --- | --- | --- | --- | --- | --- | --- | --- | --- |
| Energy (kcal) | 0.2 | 0.4 | 10.0 | 100.0 | 4.5 | 99.8 | 0.0 | 99.6 |
| Protein (g) | 0.0 | 0.0 | 8.8 | 99.9 | 0.0 | 96.9 | 0.0 | 97.0 |
| Vitamin A(µg RAE) | 0.0 | 32.5 | 0.0 | 44.6 | 25.7 | 16.5 | 7.1 | 12.0 |
| Ascorbic acid (mg) | 0.1 | 0.5 | 0.0 | 50.2 | 2.9 | 42.6 | 0.9 | 15.1 |
| Iron (mg) | 0.0 | 0.0 | 0.0 | 18.7 | 0.0 | 11.4 | 0.0 | 0.4 |
| Zinc (mg) | 0.0 | 0.0 | 0.0 | 55.1 | 0.0 | 39.8 | 0.0 | 25.9 |
| Calcium (mg) | 8.0 | 0.1 | 3.1 | 100.0 | 0.0 | 92.2 | 0.0 | 96.7 |
| Thiamine (mg) | 0.0 | 0.0 | 0.0 | 97.3 | 0.0 | 96.1 | 0.0 | 83.6 |
| Niacin (mg) | 0.0 | 0.0 | 0.1 | 99.1 | 0.0 | 91.2 | 0.0 | 82.7 |
| Riboflavin (mg) | 0.0 | 0.0 | 0.2 | 99.5 | 0.8 | 95.5 | 0.0 | 93.8 |

*Note: SNNP= Southern Nations, Nationalities, and Peoples' Region, BG = Benishangul-Gumuz.*

**Fig A: Food supplied (%) from production, import and export (Panel A) and import and export (x 1000 metric tons) by food groups (Panel B), 2011-2013**

**Panel A:**

**Panel B:**

**Import**

**Export**

Source: FAO Food Balance Sheets: <http://www.fao.org/faostat/en/#data> (accessed in June 12, 2018)
